# Design and implementation of a versatile magnetic field mapper for 3D volumes

**DOI:** 10.1016/j.ohx.2022.e00356

**Published:** 2022-09-02

**Authors:** Muhammad Nasir, Muhammad Shoaib, Muhammad Umar Hassan, Muhammad Sabieh Anwar

**Affiliations:** a Department of Physics, Syed Babar Ali School of Science and Engineering, Lahore University of Management Sciences (LUMS) Opposite Sector U, DHA, Lahore 54792, Pakistan

**Keywords:** Magnetometer, Linear stages, Hall probe, Magnetic resonance

## Abstract

Magnetic field mapping is an essential step in research, manufacturing, and maintenance. It helps one find out what’s under the surface of any object and identify areas of high-flux density. Our magnetic field mapper is easy-to-use and has a user-friendly interface. It comprises a triple axis Hall effect-based magnetometer, meaning that it can measure the strength of magnetic fields in 3D. It is made up of three Hall sensors which are mounted on a triple-slot sensor probe which in turn is attached to translation stages allowing motion in three dimensions. The translation in X and Y motion (independent) is from -50 to +50 mm while in Z is from -150 to +150 mm with a nominal resolution of 0.1 mm. The height of the magnetometer is 1000 mm and it’s originally designed for mapping the magnetic field of permanent magnet assemblies for low-field and mobile magnetic resonance scanners. Sometimes, you need to find out how strong the magnetic field in a particular region is or you may want to measure all the three components of the magnetic flux density to find out the homogeneity or isotropy of the field. In high resonant NMR spectroscopy, the magnetic field needs to be highly uniform and homogeneous and to assess such a field, we need a device which can measure the magnetic field, precisely, accurately and reproducibly. The proposed field mapper is useful in all of these situations. It is low cost and easy to manufacture. The maximum measurable magnetic field is ±2 T. Furthermore, all the material required for building this device is easily accessible.

## Graphical abstract

**Figure:**
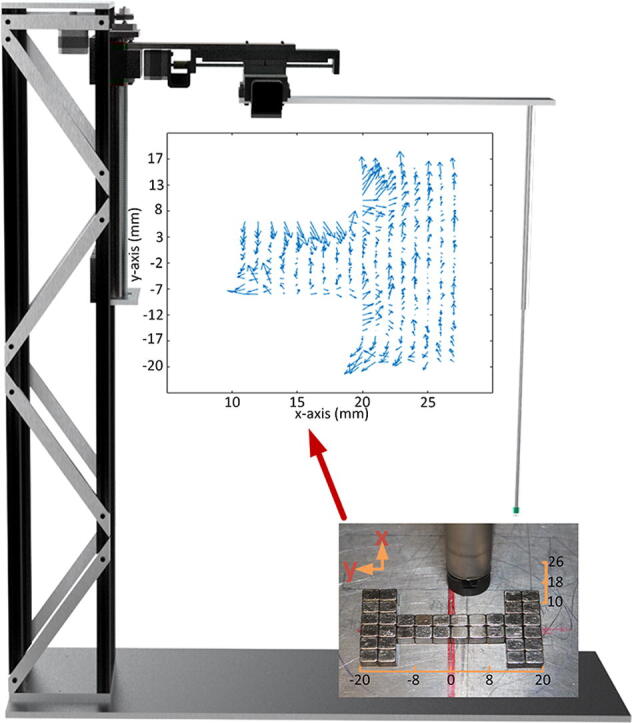

**Specifications table**

**Table:** 

**Hardware name**	Magnetic field mapper (MFM)
**Subject area**	Engineering and Material Science
**Hardware type**	Imaging and measurement tool
**Closest commercial analog**	3D magnetic field camera by Magcam
**Open source license**	CC BY 4.0
**Cost of hardware**	$424
**Source file repository**	https:/doi.org/10.17632/7jzgfwhznn.3

## 1 Hardware in context

The Magnetic Field Mapper (MFM) is a robotic sensor that uses a triple-axis magnetometer to map out large areas for magnetic field distribution. The mapping can then be analyzed to find any sources of magnetic interference, imperfections, and perform comparisons with a target field and inhomogeneity. The importance of accurate and quick measurement of permanent magnets’ fields cannot be underestimated in research, development and industrial production processes, medical devices, automobiles, motors and electronic design and manufacturing. In some applications such as NMR spectroscopy, extremely high accuracy is required in measuring the field of the NMR magnet to identify homogeneous regions which become candidate sweet spots for high resolution spectroscopy [1], [2]. In most cases, 3D mapping is also needed. This distribution of the magnetic field is measured by a Hall probe composed of Hall sensors in three dimensions [3], [4] which are then connected to translation stages which allow motion in three dimensions [5], [6], [7].

**Fig. 1:**
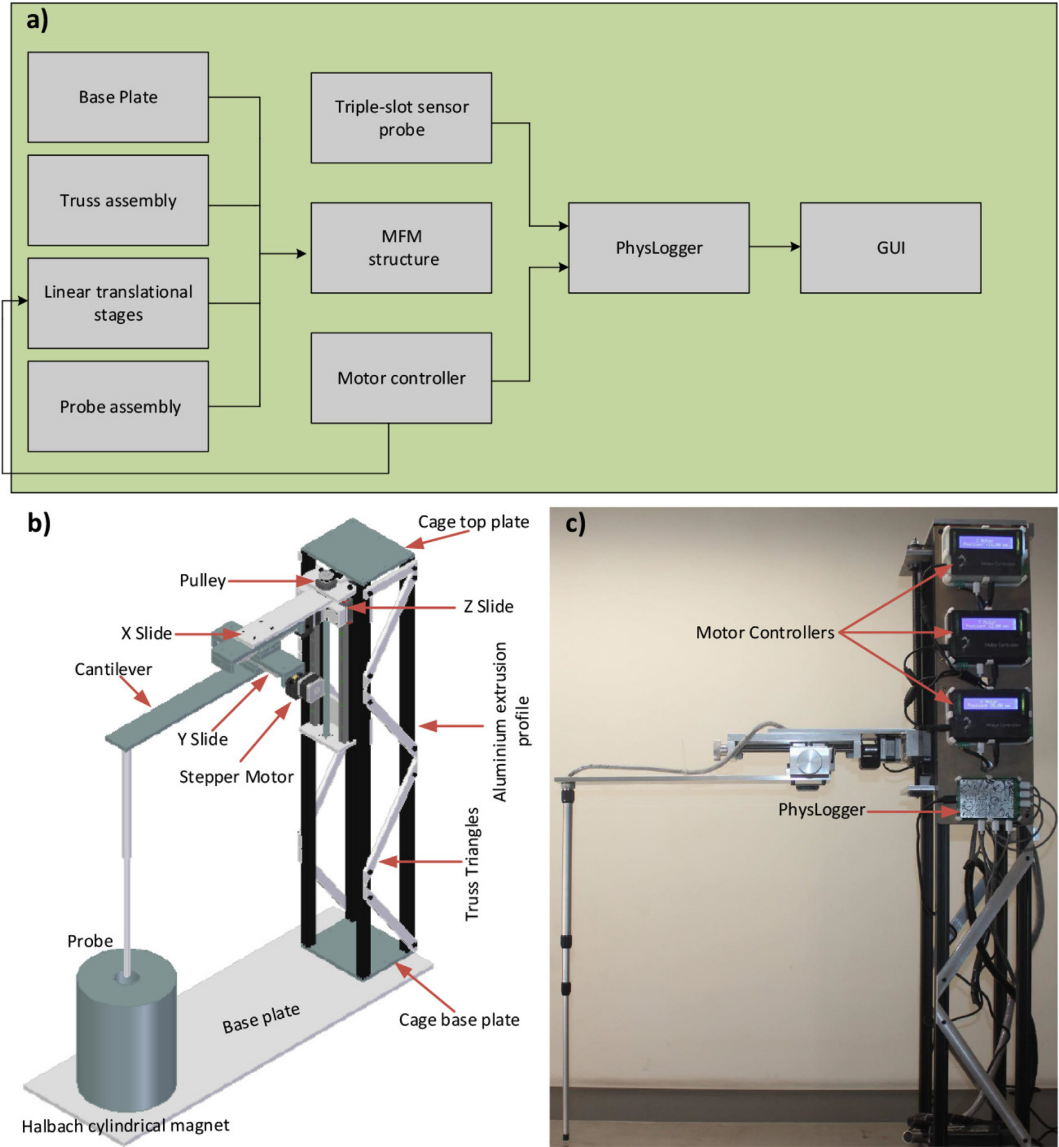
a) Block diagram illustrates how the different components integrate the MFM device. b) The basic 3D mechanical structure design of the magnetic field mapper with the three slides, truss structure, sensor probe, base plate and Halbach cylindrical magnet. c) Implemented magnetic field mapper with PhysLogger (DAQ device) and motor controllers.

Some prior solutions proposed by other works cover very small areas [8]. Commercially available mappers also measure up to ±2 T but their designs are proprietary [9], [12]. The geometry used in our proposed MFM is simple and supported by a simple truss structure. The mechanical structure is easy to build and capable of measuring the magnetic field up to ±2 T at about the same precision as other commercially available devices. It is low-cost and easily reproducible. The structure can be activated either manually or programmatically. The three linear stages are constructed on an aluminum plate called the base plate. The linear stages are 600 mm above the base plate, ensuring no ferromagnetic material is in close proximity to the sample space, and are supported by the truss mechanism to make the whole system stable during movement (Fig. 1(b)).

The approximate magnetic field sensing area is 100×100×300mm. The X, Y and Z movements of the mapper are controlled by homemade motor controllers. Theses controllers can work independently or can be controlled by a user interface using any data acquisition (DAQ) card. For MFM, we use a homemade DAQ system called PhysLogger. The PhysLogger interface not only activates the stepper motors but also records the triple-axis magnetic field measurement. Details of the control and data recording can be seen on PhysLogger’s website [16], [17].

## 2 Description

The whole apparatus is constructed on an 8 mm thick non-magnetic aluminium plate (called the base plate) of size 910×295 mm. A cage of four aluminium extrusion profiles each of cross section 20×20 mm and height 1 m is made as a stand for the Z slide as we have to keep the motors away from the sensor probe. The sensor is mounted on a triple axis translation stage which moves the sensor probe. To ensure structural stability, the trusses are assembled at an angle of 55° w.r.t. the horizontal level of the cage. Three dimensional linear stages are produced by components commonly used in a typical 3D printer such as timing belts, stepper motors, linear rails, lead screws, linear rods and bearings. For example, the Z stage is driven by a lead screw of pitch 6 mm and a timing belt while X and Y stages are driven by lead screws of pitch 8 mm. The translation and triple-axis field sensing is all computer controlled. The complete assembly is shown in Fig. 1(c). A power supply (12 V 5 A) is used to energize the motor controllers.

## 3 Design files

The design files and animated videos are located at https:/doi.org/10.17632/7jzgfwhznn.3 whose description is given in Table 1.

**Table 1:** Design files and videos of the MFM.

Design file name	File type	Open source licence
Solid Edge MFM assembly file	step file (.stp)	CC BY 4.0
zip folder containing Solid Edge files	.prt and.asm	CC BY 4.0
zip folder containing drawings of the machined parts	.pdf	CC BY 4.0
Magnetic probe housing	.stl file	CC BY 4.0
Motor mounting coupling	.stl file	CC BY 4.0
Truss.wmv	.wmv	CC BY 4.0
Z slider assembly.wmv	.wmv	CC BY 4.0
Z slide assembly.wmv	.wmv	CC BY 4.0
X or Y Slide.wmv	.wmv	CC BY 4.0
X and Y Slide.wmv	.wmv	CC BY 4.0
Mounting Z slide on the truss.wmv	.wmv	CC BY 4.0
Final Assembly.wmv	.wmv	CC BY 4.0
Probe sensor.wmv	.wmv	CC BY 4.0
mfm video.mpeg	.mpeg	CC BY 4.0

### 3.1 3D structure files

The complete assembly of the MFM is located in the step (.stp) file which can be opened in any open source CAD 3D software. Once it opens, all components files will automatically open and become immediately accessible. All the aluminium plates, slides, triangle and rectangular strips and base parts are manufactured on a milling machine. The drawings of the machined parts are also included in a zip folder. The Hall sensor housing is manufactured with a (Creality) resin printer whose.stl file is also situated in the data repository.

### 3.2 Videos

The animated videos (.wmv files) recording the phased, stage-wise construction of the assembly are also located in the given repository location which also contains working video (.mpeg file) demonstrating the GUI and recording of data.

## 4 Bill of materials summary

The bill of materials (BOM) has two subsections, one for machined components and other for the purchased components. The designator column in the machined components table directs the reader to the repository. For the brevity, “Drawings of the machined parts.zip” folder has been relabeled as “Drawings” in the designator column. All of these components were purchased off-the-shelf from a local market in Lahore and are generally available in any standard mechanical workshop. The “source of materials” column in the purchased components table includes source for off-the-shelf purchasing.

### 4.1 Machined components

.

**Table:** 

**Designator**	**Component**	**Number**	**Cost per unit** $	**Total cost** $	**Material type**
Drawings >> Base Plate.pdf	Base plate (910×295×8 mm)	1	44.00	44.00	aluminium
Drawings >> X or Y Slide >> H shape.pdf	H shape assembly	2	7.40	14.80	aluminium
Drawings >> Truss >> Truss Triangles.pdf	Truss triangles strips (261×20×10)	8	1.50	12.00	aluminium
Drawings >> X or Y Slide >> Slide Base.pdf	Base plate for X and Y stages (213×60×12 mm)	2	4.5	9	aluminium
Drawings >> Z Slide >> Joint Plate.pdf	Plate (180×65×8 mm) to joint Z slide with truss	2	4.5	9	aluminium
Drwaings >> Cantilever.pdf	Cantilever aluminium plate (454×60×8 mm)	1	5.20	5.20	aluminium
Drawings >> Z Slide >> Slider Assembly.pdf	Z slider assembly	1	3.71	3.71	aluminium
Drawings >> Joint Plate XY&Z.pdf	Plate (265×60×8 mm) to joint X, Y and Z slide	1	3.70	3.70	aluminium
Drawings >> X or Y Slide >> Side Plate.pdf	Side plates for X and Y slides (60×22×12 mm)	4	0.65	2.60	aluminium
Drawings >> Truss >> Rectangular Strip.pdf	Truss rectangular strips (150×20×10 mm)	2	1.00	2.00	aluminium
Motor mounting coupling.stl	3D printed mounting assembly for motor coupling	2			PLA
Magnetic Probe Housing.stl	3D printed Hall senor housing	1	0.5	0.5	UV resin

### 4.2 Purchased components

.

**Table:** 

**Component**	**Number**	**Cost per unit** $	**Total cost** $	**Source of Materials**	**Material type**
Motor controller	2	25	50	physlogger.com	
PhysLogger	1	40	40	physlogger.com	
Extrusion profiles of length 1 m	4	10.00	40.00	Suggested: 2020 T-Slot (amazon.com)	aluminium
Lead ball screw	1	32.80	32.80	Suggested: SFU1006 (amazon.com)	stainless steel
Linear guide rails (15 mm diameter and 370 mm length)	2	13.66	27.32	Suggested: MS-2-HGH15CA-L640MM (amazon.com)	carbon steel
M4 cap head screws of length 15 mm	54	0.40	21.60	Suggested: 9180623 (amazon.com)	alloy steel
Linear guide rails (9 mm diameter and 180 mm length)	2	8	16	Suggested: 6725K43 (mcmaster.com)	stainless steel
C-type cables of length 610 mm	6	2	12		
Ball bearings	2	5.50	11.00	Suggested: FK12 (damencnc.com)	iron
Extrusion profiles of length 375 mm	2	4.70	9.40	Suggested: 2020 T-Slot (amazon.com)	aluminium
Hall Sensor	3	1.85	5.55	Suggested: CYSJ902 (sonnecy-shop.com)	
Rod of adjustable length (300 to 1000 mm)	1	5.40	5.40		aluminium
Lead screw (8 mm diameter)	2	3.80	3.80	Suggested: Tr8x8 (amazon.com)	stainless steel
Flexible shaft coupling (8×5 mm)	2	1.90	3.80	Suggested: 4147N168 (mcmaster.com)	aluminium
DC power adapter (12 V 5A)	1	3.66	3.66	Suggested: 60 W 12 V 5A AC-DC Power Adapter (amazon.com)	
Pulley having 40 teeth	1	3.50	3.50	Suggested: 0696735285401 (amazon.com)	metal
M3 cap head screw of length 15 mm	68	0.04	2.72	Suggested: 91292A115 (mcmaster.com)	stainless steel
Timing belt	1	2.20	2.20	Suggested: S2M-202 (amazon.com)	rubber
M4 button head screw of length 15 mm	30	0.05	1.50	Suggested: 97763A425 (mcmaster.com)	alloy steel
Ball screw nut (10 mm diameter)	1	0.82	0.82	Suggested: MS-SFK10mm (amazon.com)	carbon steel
Pulley having 18 teeth	1	0.50	0.50	Suggested: M-GT2-PULLEY-18T (amazon.com)	metal
Ball screw nut (8 mm diameter)	1	0.80	0.80	Suggested: UF-ZX20-TD5K (amazon.com)	carbon steel
M5 tapered head screws of length 20 mm	4	0.05	0.20	Suggested: 92125A214 (mcmaster.com)	stainless steel

## 5 Build instructions

Fig. 1 shows the basic assembly of the MFM which is designed and constructed using the concept of three Cartesian linear stages of the 3D printer. The X slide holds the magnetic sensor probe cavity which is 700 mm away from the truss cage structure. The X slide is mounted on an orthogonal Y slide which in turn connects to a Z slide. These slides are fixed on the truss structure of 1 m height to avoid any ferromagnetic material, within the structure, near the probe. Finally, the entire truss structure rests on an aluminum base plate. We now describe the various segments of the MFM, outting how the entire structure can be built.

### 5.1 The truss assembly

**Fig. 2:**
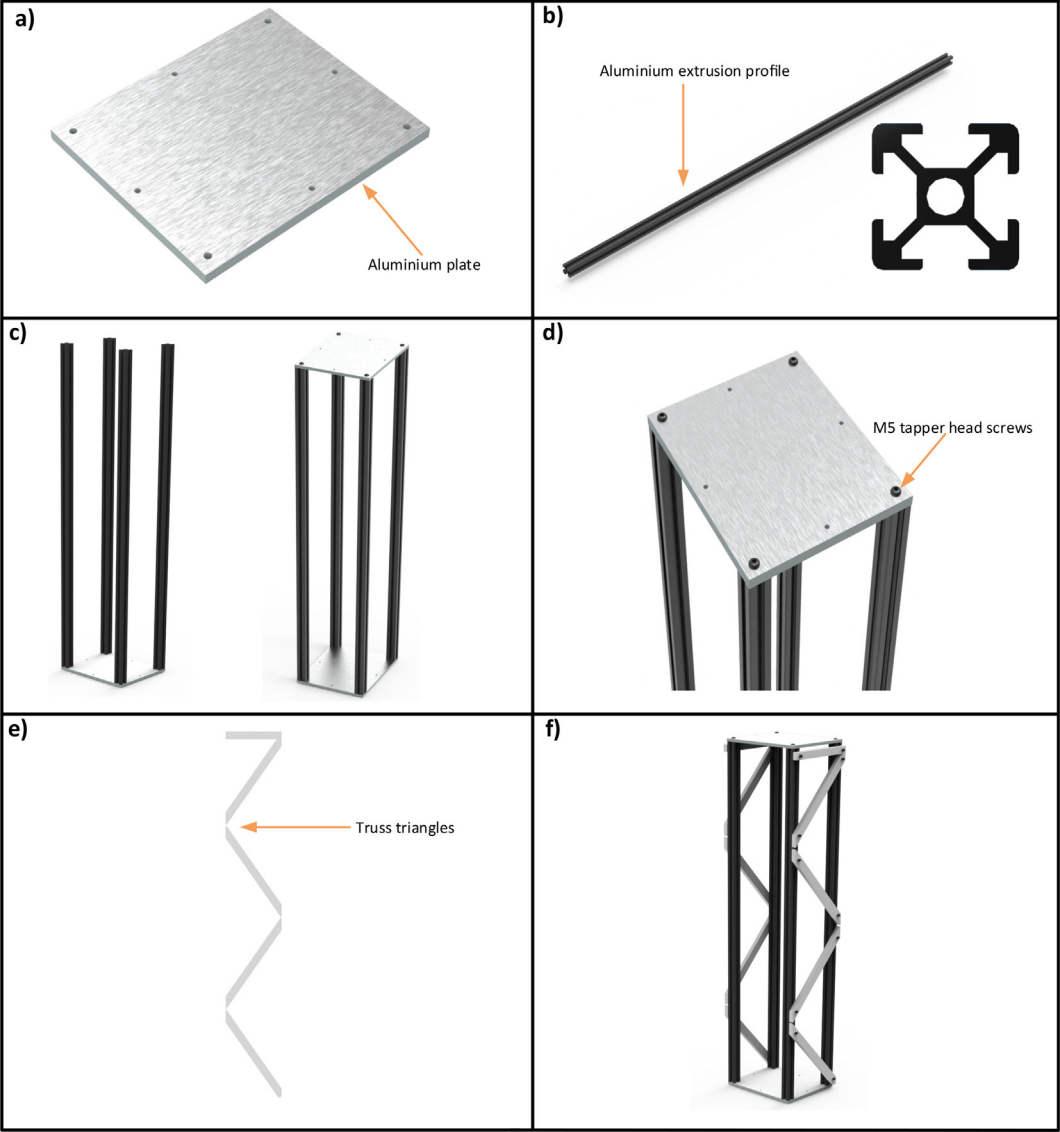
The truss structure in steps. a) base plate for truss, b) aluminium extrusion profile (20×20 mm) of length 1 m and the cross sectional view of it, c) the completion of truss box, d) a close view of with the tightened screws, e) the side and front view of the truss triangles and f) the complete truss structure.

The truss provides stability to the MFM. It is produced quickly and cheaply as it needs no formwork to support its shape. The truss is connected in triangles and behaves like a single solid object. The truss is built using four aluminium extrusion profiles of cross section 20×20 mm and length 1000 mm respectively (see Fig. 2(b). The cross section is a hollow square with diagonal protrusions outside the square, terminating in the vertices of another large square. These four profiles are mounted on an aluminium plate (called cage base plate) (Fig. 2(a)) of size 180×150×8 mm using M5 tapered head screws. A similar aluminium plate is placed at the top of these profiles to complete the cage as shown in Fig. 2(c) and 2(d). Fig. 2(e) describes the triangles of the truss that are constructed by aluminium strips of dimensions 20×10×260 mm. These strip are cut by a milling machine at an angle of 55° from both the ends. Five of these strips cover one side of the cage. To form the triangles, the first strip is placed at an angle of 55° from the surface and the second strip is placed in such a way that the angle between the two strips is 110°. The triangles are made on only two opposite sides of the cage. The complete truss structure can be seen in Fig. 2(f). The Z slide is placed on an untriangled side. All these strips are mounted on the cage ribs by M4 cap head screws. An animated video of the construction of the truss and the drawings of the machined parts is available in the data repository linkhttps:/doi.org/10.17632/7jzgfwhznn.3 (Truss.wmv, Drawings of machined parts >> Truss).

### 5.2 The Z slide

**Fig. 3:**
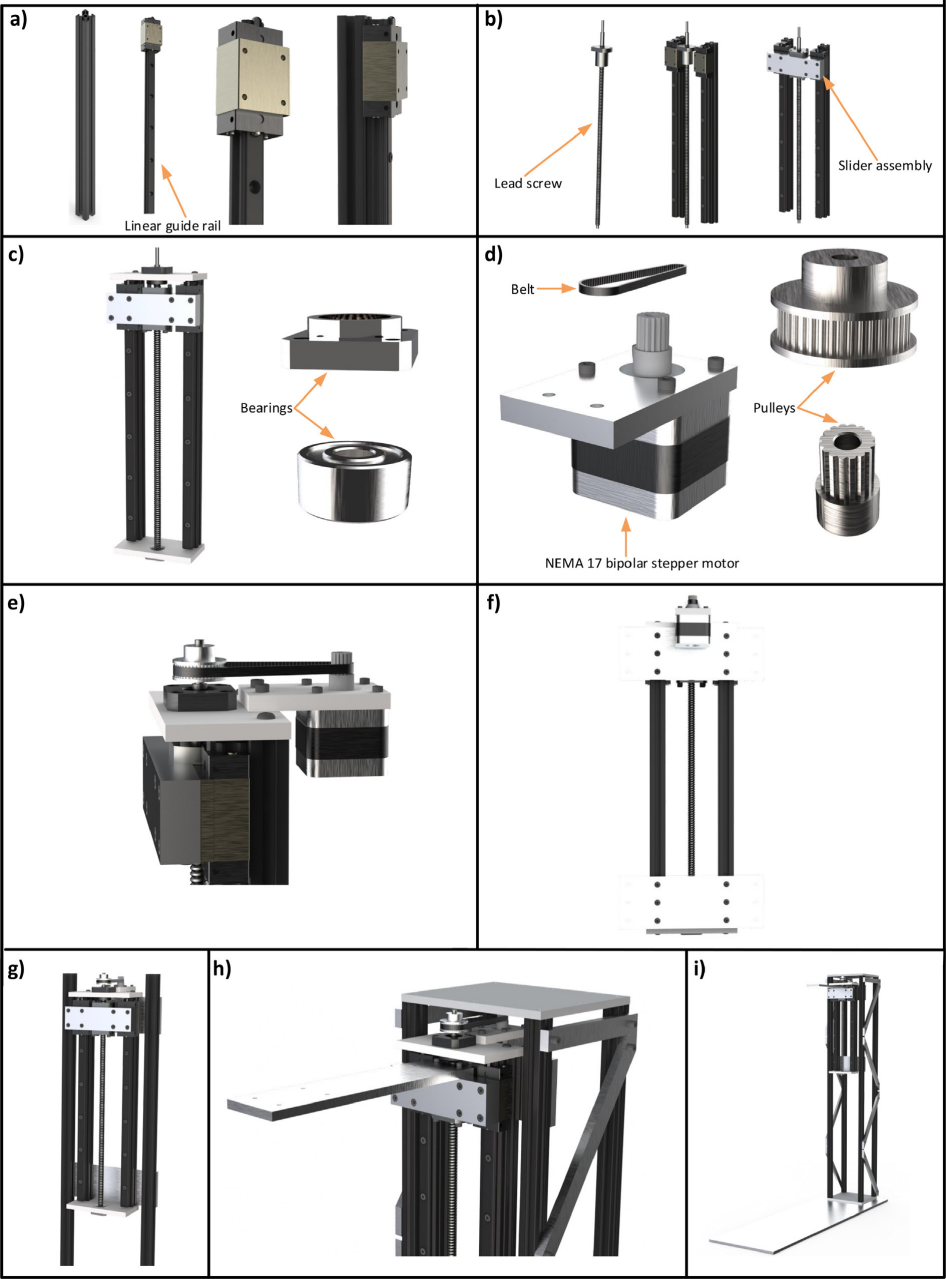
Step by step assembly of the Z slide, a) 375 mm long aluminium extrusion profile (20×20 mm) with linear guide stage, b) assembling lead screw with linear guide stages, c) mounting the structure on the aluminium plates with bearings, d) components of the motor coupling, e) view of the stepper motor after adjusting with the Z slide, f) the complete Z slide after attaching all the components, g) attachment of the Z slide with the truss structure, h) close up view of the structure and i) view of the mapper after attaching the Z slide.

Two aluminium extrusion profiles (20×20 mm) of length 375 mm, two linear guide rails (15 mm), lead screw (length 440 mm, diameter 10 mm, pitch 6 mm), two ball bearings (FK-12), one ball screw nut (10 mm), two top and bottom plates of aluminium (115×60 mm), aluminum assembly of the slider and two joint plates are configured to construct the Z slide. Both linear guide stages are mounted on the aluminium extrusion profiles being 66 mm apart from each other (see Fig. 3(a) and 3(b)). The linear guide stage and lead screws are joined together with the slider assembly and a ball screw nut, as can be seen in Fig. 3(b). Finally, M3 cap head screws hold these assemblies. Two aluminium plates (115×60 mm) shown in Fig. 3(c) also support this assembly from the top and bottom by ball bearings. The allowable distance travelled by the Z slide is 300 mm. The slide is translated by a NEMA 17 stepper motor which is attached to the slide with a timing belt (S2M 202) and pulleys with 18 and 40 teeth respectively. These are shown in Fig. 3(d) and 3(e). The gear ratio of this assembly is therefore (18/40)×6=2.7 where 6 is the screw pitch. A close up view of the back side is shown in Fig. 3(f).

This assembly is fastened to the truss structure with two aluminium plates (180×65×8 mm) from top and bottom which is described by Figs. 3(f) and 3(g). The X and Y slides are also mounted on the Z slide at this slider assembly with the help of an aluminium plate (265×60×8). Fig. 3(h) describes these assemblies together while the overall structure incorporating the Z slide is shown in Fig. 3(i). The animated videos of the construction of the Z slide and the drawings of the machined parts are available in the repository https:/doi.org/10.17632/7jzgfwhznn.3 (Z slider assembly.wmv, Z slide assembly.wmv, Mounting Z slide on the truss.wmv, Drawings of machined parts >> Z Slide).

### 5.3 The X and the Y slides

**Fig. 4:**
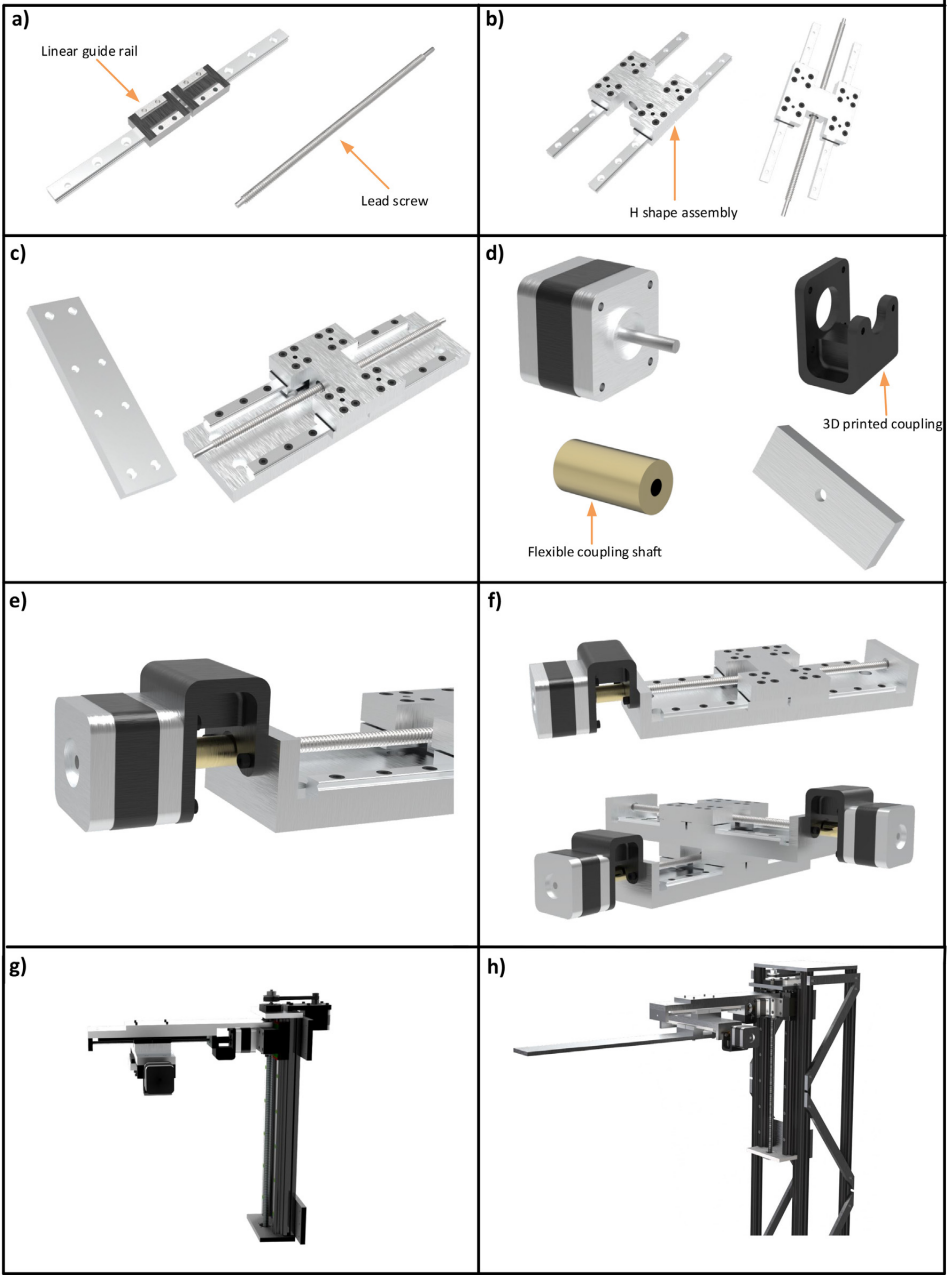
X and Y slides stages, a) linear guide stage and the lead screw, b) assembling the linear stages and lead screw with the help of H shape assembly, c) assembly is mounted on the 12 mm thick aluminum plate (213×60), d) motor assembly components, e) assembling the motor coupling with the slide, f) X slide is mounted on the Y slide, g) attaching the X and Y slides with Z slide and h) close up view of all the three slides with the truss structure.

In order to construct X and Y slides, two linear guide stages (9 mm), each possessing two sliders, are mounted on an aluminium plate (213×60×12) with M3 cap head screws. These linear guide stages are 40 mm apart. A lead screw (8 mm) and linear guide stage are assembled together by an H shaped assembly and a screw nut (8 mm). This entire process is illustrated in Fig. 4(a) through 4(c). Both ends of the lead screw are fixed by aluminium plates (60×22×12 mm). One end of the lead screw is attached with a knob using a bearing for manual movement of the stage while the other end is attached with the NEMA 17 stepper motor with flexible coupling shaft (5×8 mm) and an assembly, which can be seen in Fig. 4(d) to 4(f). Moreover, M3 cap head screws are used for fastening the coupling assembly. Both X and Y slides are assembled with the same components and have identical dimensions and sizes. The movement in either the X or Y slide is 110 mm. The gear ratio in these stages is 8 mm as there are no timing belt and pulleys in these stages and the gear ratio is equal to the pitch of the screw lead. The Y slide is attached below the X slide with the H shape assembly part while the magnetic probe assembly is attached with the H shape assembly part of the Y slide. After assembling both of these slides, they are mounted on the Z slide through a connecting aluminium plate. See Fig. 4(f), 4(g) and 4(h). Once again, animated construction videos are in our repository. (X or Y slide.wmv, X and Y slide.wmv, Drawings of machined parts >> X or Y Slide, Drawings of machined parts >> Joint Plate XY&Z.pdf).

### 5.4 The probe assembly

**Fig. 5:**
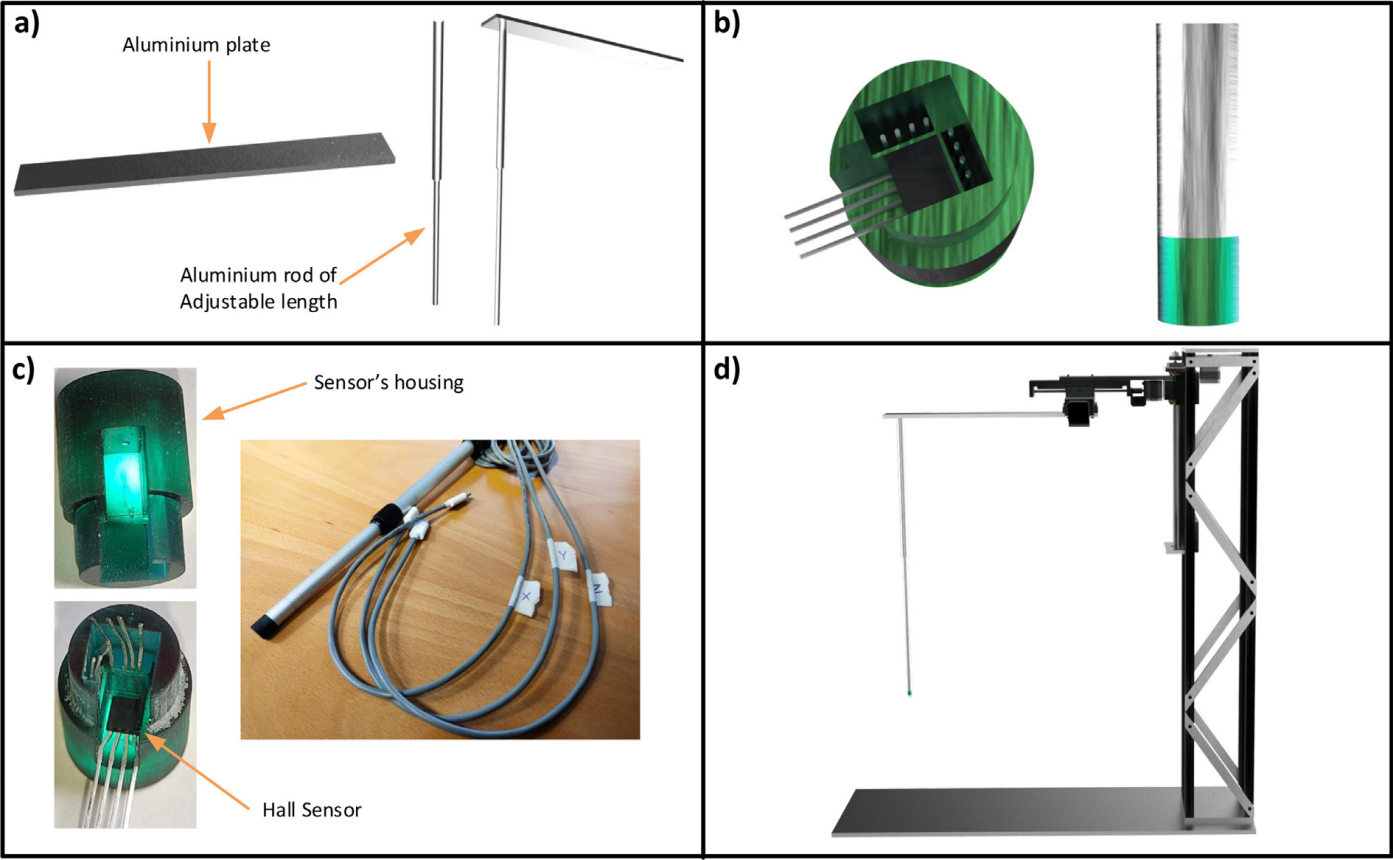
The completion of the probe assembly, a) aluminium rod of adjustable length and an aluminium plate (454×60) of thickness 8 mm for connecting the probe with the entire structure, b) the designed 3D structure of Hall sensor housing with sensors and the attachment with the rod, c) the printed 3D housing with the sensors and the complete manufactured magnetic hall probe and d) the complete structure of the magnetic field mapper.

Fig. 5(a) describes the assembly of the magnetic field probe, which is styled as a cantilever, probe face down, consisting of an aluminum plate (455×60×8), an aluminum rod of minimum height 235 mm and maximum height 1000 mm, which is hollow from the inside and whose length is adjustable. It has a 3D printed sensor housing comprising pockets for each axis. The sensor housing holds Hall sensors (CYSJ902 GaAs Hall Effect Element) in the X, Y and Z directions which measure the respective magnetic fields. The 3D housing is designed in Solid Edge and made on a Creality resin printer and cured by ultraviolet (UV) light. Fig. 5(b) describes the designed sensor housing. After snug placement of the sensors to the respective pockets, the housing is inserted into the hollow aluminium rod whose inside and outside radii are 8 and 10 mm see Fig. 5(c). An adapter is designed and manufactured by turning with threaded M16 on both the sides to attach the aluminium rod to the plate. The plate is then attached to the Y slide H shape assembly with M4 cap head screws see Fig. 5(d). An animated video of final assembly and drawings of machined parts are available in the dataset link https:/doi.org/10.17632/7jzgfwhznn.3 (Probe sensor.wmv, Final assembly.wmv, Drawings of machined parts >> Cantilever.pdf, Drawings of machined parts >> Base Plate.pdf).

### 5.5 Motor controller

To manually control the stepper motors driving the linear stages, we use a home-grown, open-source stepper motor controller device [13]. This device is equipped with a TMC2208 smart motor controller which is controlled using an STM32F103C8T6 powered, Arduino-based development board, also called “The Blue Pill” [14], [15]. With the help of an alphanumeric liquid–crystal display (LCD) screen and push buttons, the microcontroller provides a user interface to input basic controlling parameters such as the mechanical transfer functions, movement speed, and position. The motor controller is also compatible with PhysLogger, which is used to integrate multiple linear stages and for providing a more robust, computer-based user interface [16].

### 5.6 Data acquisition and control

To digitally control the linear stages and acquire and record the sensor measurements, we use PhysLogger, which is a pocket-sized data logging device with robust hardware and a friendly user interface [16]. Primarily, PhysLogger lets us measure and control voltage on multiple physical channels but it can also interface with a family of instruments that can measure and control other derived quantities. One of these instruments is called a Stepper Motor Controller that can drive bipolar stepper motors and another is called a PhysHall which is a magnetic flux sensor equipped with the GaAs Hall effect detection chip [16]. Once connected with the PhysLogger, the user interface allows us to talk to the sensors and actuators with intuitive on-screen widgets that can be customized according to the job requirements. One of these widgets is connected to two of the motor controllers to manually control the movement of the sensing probe in two dimensions. Another widget is an on-screen data table that records the position of all the linear stages as well as the magnetic field vector components corresponding to a particular three-dimensional position. Once measurement data has been collected on enough physical positions in the vicinity of an assembly of magnets, the data table is exported to any open source data processing software for further analysis.

## 6 Operation instructions

**Fig. 6:**
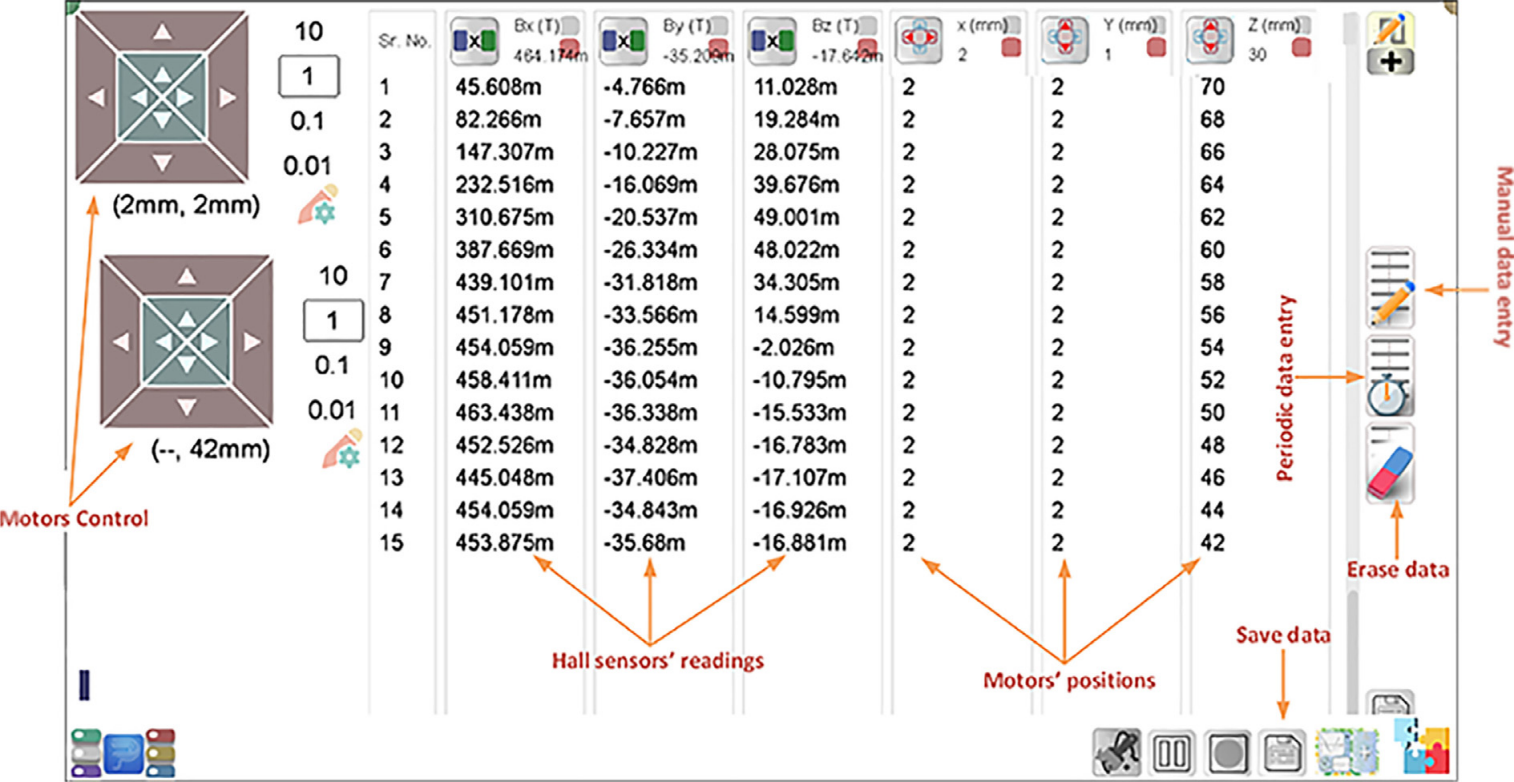
GUI interface for the MFM. Different sections are shown such as motor control, Hall sensors’ reading of the three axis and the position of the motors. The suffix ‘m’ denotes millis ($\times 10^{-3}$).

Motor controllers are powered up by a 12 V and 5 A direct current (DC) adapter. All the three motor controllers are derived by the same supply. PhysLogger is connected to the PC by using a C-type USB cable. The motor controllers are connected with the digital pins of the PhysLogger by C–C type USB cables. The Hall sensors are connected at the analog channels of the PhysLogger. The user interface is populated by selecting several widgets from the graphical menu to graphically control the motor controllers and collect data. This process involves renaming Hall sensors and motor controllers to match the printed labels on the devices, adjusting gear ratios, pitch of the linear slides, and configuring channels that correspond to the respective Hall sensors. Once the linear stage widgets have been set up to work with the motor controllers, the magnetic probe can be moved in any direction in controlled steps. It means that the probe can be moved to any three dimensional position in the work space where we want to measure the magnetic field. The stages can be moved in fine or coarse steps as listed in the linear stage widget and can be seen in Fig. 6. In parallel, a data table widget is also added to the same work space which has a data column for three hall reading and its three dimensional. The data can be acquired in this data table containing the three axis movement positions of the motors and their corresponding magnetic field in the three dimensions. The data can be entered manually by clicking a manual data entry button or periodically by setting a sampling period. After collecting data, it can be exported for further analysis to Matlab or any other data processing software. The operation of the magnetic field mapper can be seen in the given video link in Table 1. Besides the working of the MFM, there are some important safety instructions which are also listed below.

• There should be no ferromagnetic material near the MFM while taking the readings as they can change the values of magnetic field.
• Do not touch the sensor probe or the magnet which is under test while performing experiments for accurate readings.
• Use the MFM on a horizontal surface.
• Avoid exposing the Hall probe to the direct sunlight or to strong light source.

## 7 Results and discussion

### 7.1 Results

**Fig. 7:**
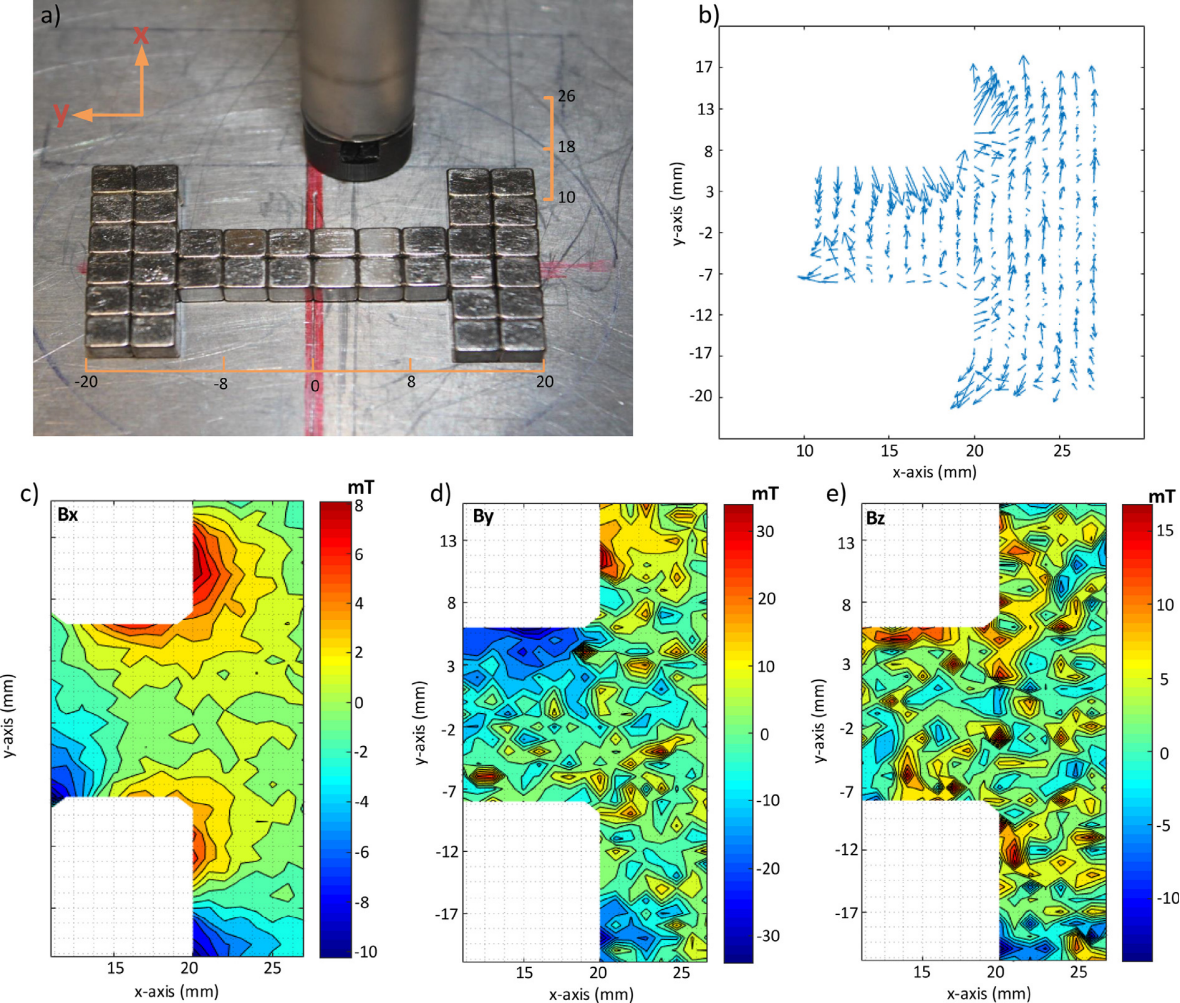
a) MFM three dimensional Hall probe sensor is utilized to measure magnetic field components of an I-shaped assembly of magnets, orange lines are showing the scale bar of the measured region. b) quiver plot to show the magnetic field lines of the measured area. c), d) and e) describe the magnetic field distribution along $B_{x},B_{y}$ and $B_{z}$ respectively.

In order to test and validate this device, the magnetic field of different shaped magnets is measured. The first example is of an I-shape assembly which is formed by assembling small cubic magnets. The assembly and its dimensions are shown in Fig. 7(a). The magnetic field is measured along one side of the magnet. The quiver plot in Fig. 7(b) shows the direction of the magnetic field in the xy plane through directed arrows. Furthermore, the results of the three magnetic field components are shown in Fig. 7(c), (d) and (e).

**Fig. 8:**
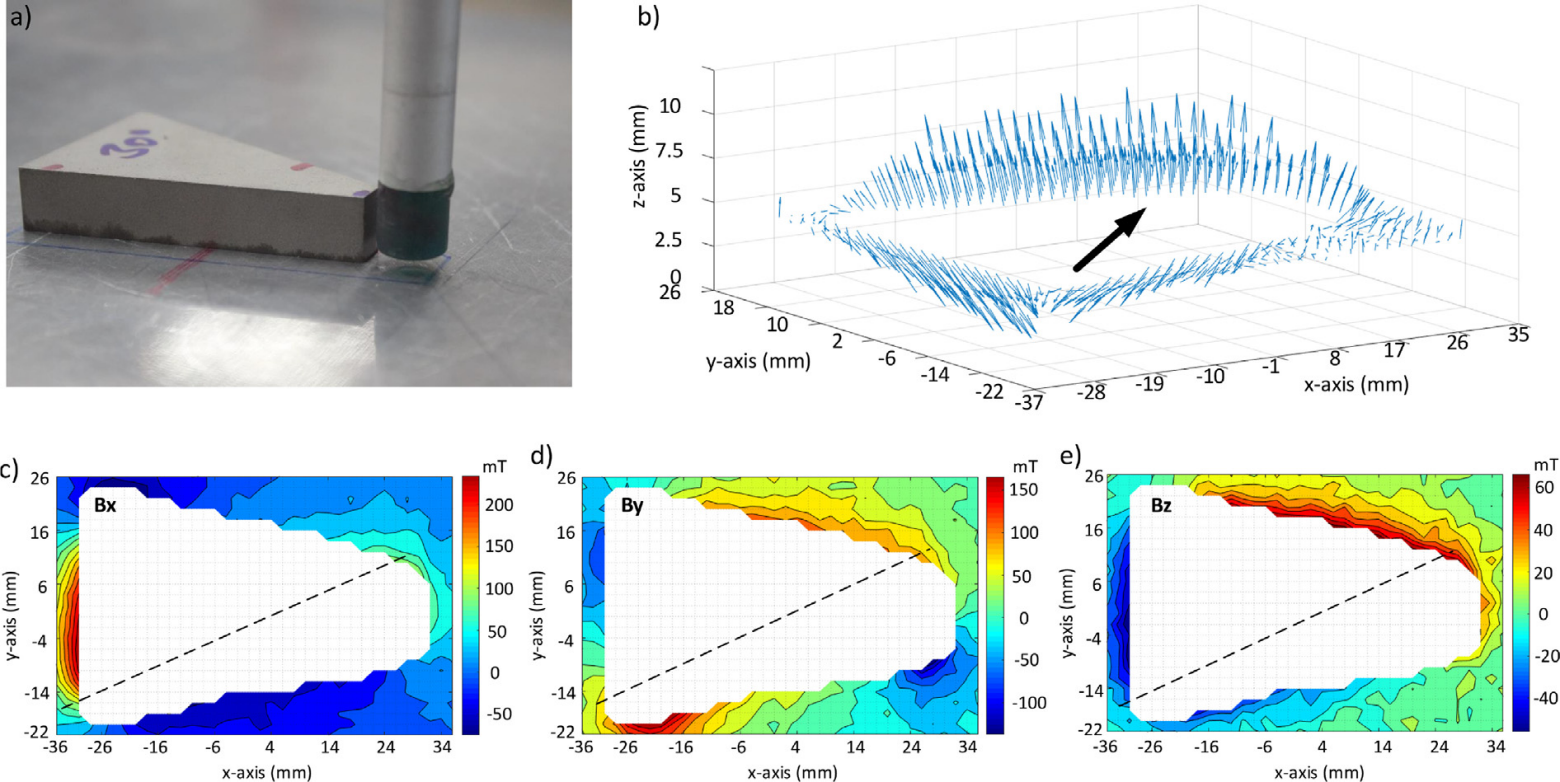
a) The trapezium magnet. b) magnetic field lines in the 3D plane, with the black arrow or the dashed line representing the manufacturer-specified magnetization direction c), d) and e) describe the magnetic field distribution along the three vectorial directions.

The second example is of a trapezoidal magnet which is magnetized at an angle of 30° w.r.t. the central axis of the magnet and shown in Fig. 8(a). The parallel sides of the magnets are 31.66 mm and 5 mm while the slanting sides are 50 mm each. The magnet is placed on the MFM base plate and the field is measured in a rectangular region outside and surrounding the magnet body whose area is 70 mm × 50 mm. The field is measured after every 2 mm. Fig. 8(b) shows the three dimensional magnetic field lines as a quiver plot. The thick arrows identifies the magnetization direction of the magnet which is 30°. Fig. 8(c), (d) and (e) are respectively showing the magnetic field distribution along x,y and *z* directions within our region of interest.

**Fig. 9:**
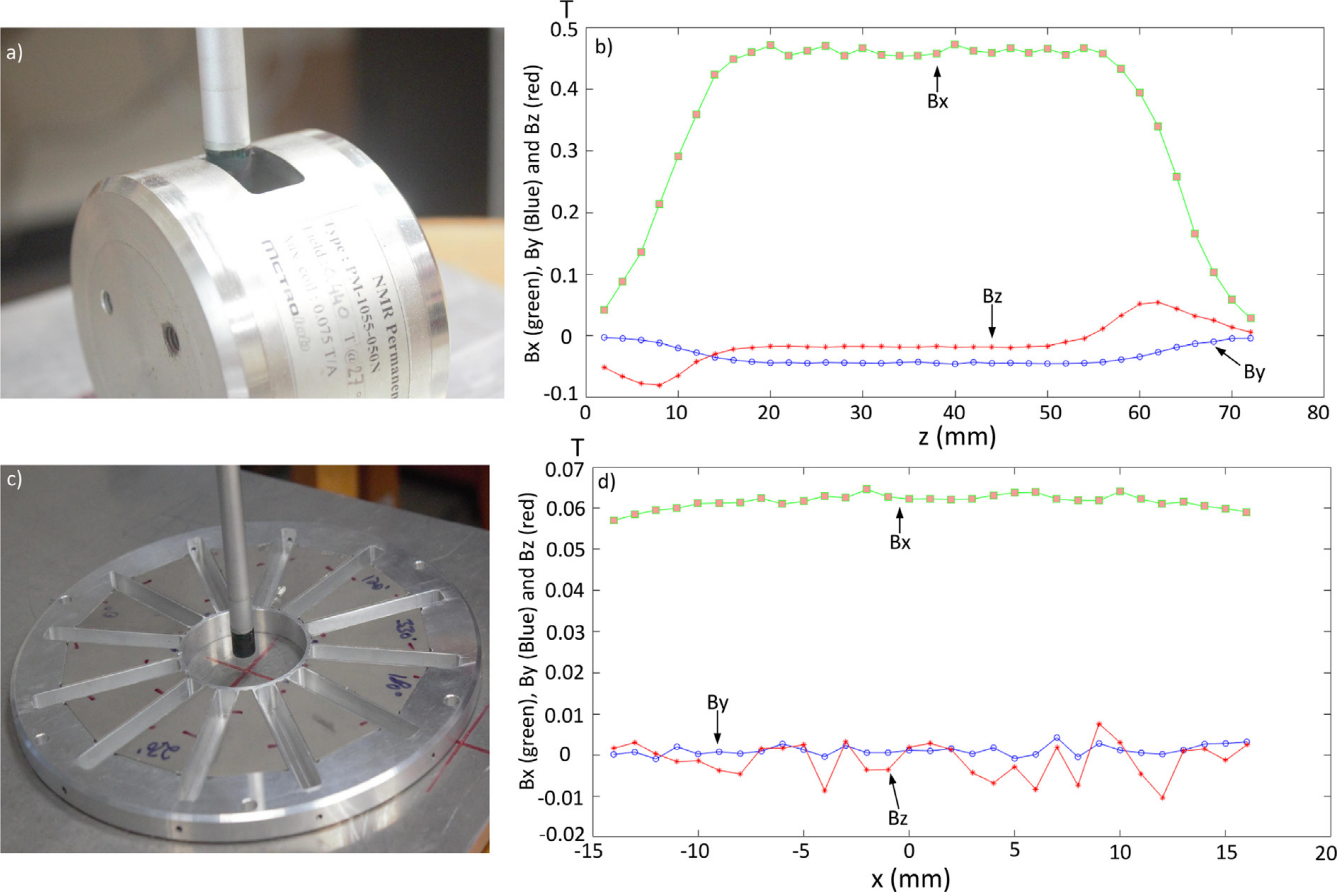
a) An NMR permanent magnet in relation to the MFM probe. b) The magnetic field components along three axis as the probe move along the z-axis with 0 to 72 mm and at the fixed setting of x and y. c) A Halbach ring magnet, d) the magnetic field along x, y and z axis components which are measured by the MFM. The plot is shown at a fixed value of y and z which is zero and x varies from -15 to 15.

The third example is an NMR-specific permanent magnet. Fig. 9(a) shows the magnet (PM-1055–050 N, Metrolab) which has a nominal field of 0.45 T. The movement of the probe is along the z-axis and the field is measured every 2 mm. The total distance covered by the probe inside the magnet is 72 mm. The probe measures the three dimensional magnetic field at each point. The graph in Fig. 9(b) shows the vectorial field indicating a region of high field uniformity with a top-hat profile.

The fourth example is a ring of trapezoidal magnets. This arrangement is a variant of the Halbach design [10] and can be employed in low-field NMR systems [11]. The goal is to keep the magnetic field horizontal and homogeneous in a small region where the sample whose NMR spectrum is to be acquired, is placed. A single Halbach ring is shown in Fig. 9(c) and a graph of the magnetic field inside the ring is shown in Fig. 9(d). The three dimensional sensor of the MFM measures three components of the magnetic field at each point inside the ring every 1 mm distance. The inside diameter of the ring is 30 mm. The graph is plotted at fixed values *y* and *z* which are zero while *x* varies from -15 to 15 mm.

### 7.2 Discussion

The field of different magnetic shapes include I-shaped assembly, trapezoidal magnet, a halbach ring and an NMR permanent magnet is measured with the help of our constructed MFM. The results obtained by MFM are highly accurate and reliable. The results obtained from MFM are either according to the datasheet, or in accordance with simulated profiles (with 0.5% error). Principally, the proposed MFM can measure any shape or volume magnet which has dimension under 100×100×300 mm, with a spatial resolution of 0.1 mm in each axis and magnetic field up to ±2 T, with selectable full-scale ranges of ±1.5 T, ±1 T and ±100 mT, with field resolutions of 5 mT, 0.5 mT and 0.05 mT respectively.

## Declaration of Competing Interest

The authors declare that they have no known competing financial interests or personal relationships that could have appeared to influence the work reported in this paper.
